# High hepatocyte growth factor expression in primary tumor predicts better overall survival in male breast cancer

**DOI:** 10.1186/s13058-020-01266-x

**Published:** 2020-03-18

**Authors:** Si-Qi Qiu, Johan van Rooijen, Hilde H. Nienhuis, Bert van der Vegt, Hetty Timmer-Bosscha, Elise van Leeuwen-Stok, Annemiek M. E. Walenkamp, Carolien H. M. van Deurzen, Geertruida H. de Bock, Elisabeth G. E. de Vries, Carolien P. Schröder

**Affiliations:** 1 Department of Medical Oncology, University of Groningen, University Medical Center Groningen, PO Box 30.001, 9700 RB Groningen, The Netherlands; 2 Diagnosis and Treatment Center of Breast Diseases, Affiliated Shantou Hospital, Sun Yat-sen University, Shantou, China; 3 The Breast Center, Cancer Hospital of Shantou University Medical College, Shantou, China; 4 Department of Internal Medicine, Martini Hospital Groningen, Groningen, The Netherlands; 5 Department of Pathology, University of Groningen, University Medical Center Groningen, Groningen, The Netherlands; 6 BOOG Study Center, Amsterdam, The Netherlands; 7 Department of Pathology, Erasmus MC Cancer Institute, Rotterdam, The Netherlands; 8 Department of Epidemiology, University of Groningen, University Medical Center Groningen, Groningen, The Netherlands

**Keywords:** Male breast cancer, Hepatocyte growth factor (HGF), Stromal cell-derived factor-1 (CXCL12), Prognosis, Tumor biology

## Abstract

**Background:**

Breast cancer is rare in men, but management is focused on tumor characteristics commonly found in female breast cancer. The tumor microenvironment of male breast cancer is less well understood, and insight may improve male breast cancer management. The hepatocyte growth factor (HGF)/c-MET axis and the stromal cell-derived factor-1 (CXCL12)/C-X-C chemokine receptor type 4 (CXCR4) axis are prognostic in women with breast cancer. We aimed to investigate these factors in male breast cancer and correlate them with patient survival.

**Methods:**

From 841 Dutch males with breast cancer who were enrolled in the EORTC 10085/TBCRC/BIG/NABCG International Male Breast Cancer Program (NCT01101425) and diagnosed between 1990 and 2010, archival primary tumor samples were collected. Tissue microarrays were constructed with 3 cores per sample and used for immunohistochemical analysis of HGF, c-MET, CXCL12, and CXCR4. Overall survival (OS) of the patients without metastases (M0) was analyzed using the Kaplan-Meier method. The value of the markers regarding OS was determined using univariable and multivariable Cox regression analyses, providing hazard ratios (HRs) and 95% confidence intervals (95% CIs).

**Results:**

Of 720 out of 841 patients, sufficient tissue was available for analysis; 487 out of 720 patients had M0 disease. Patients with high HGF expression and high CXCL12 expression had a superior OS (low vs high expression of both markers, 7.5 vs 13.0 years, hazard ratio [HR] 0.64, 95% CI 0.49–0.84, *P* = 0.001 [HGF]; 9.1 vs 15.3 years, HR 0.63, 95% CI 0.45–0.87, *P* = 0.005 [CXCL12]). Multivariate analysis identified HGF as an independent predictor for OS (HR 0.64, 95% CI 0.47–0.88, *P* = 0.001).

**Conclusions:**

HGF and CXCL12 tumor expression appear to identify male breast cancer patients with a relatively good prognosis. Possibly, this could support male breast cancer-specific management strategies in the future.

## Background

Breast cancer in men is a rare disease. Although only 0.5–1% of all breast cancers occur in men, the incidence is slowly rising [1, 2]. Generally, male breast cancer has more favorable tumor characteristics than female breast cancer, such as lower tumor grade, a higher incidence of estrogen receptor (ER) expression, and a lower incidence of human epidermal growth factor receptor 2 (HER2) expression [1, 3]. On the other hand, male patients present with higher stages of disease at first diagnosis than women [1, 4]. Although the outcome in male breast cancer is similar compared to women after correction for age and stage, in general, survival improvement in men is still lagging behind [1, 4–7]. Due to the lack of survival data from randomized trials in male breast cancer, treatment strategies for this disease are largely based upon data from studies of treatment for female breast cancer. In recent years, it becomes clear that the male breast cancer biology may have distinct properties compared to females [8–11]. Therefore, a better understanding of the breast tumor characteristics in men may help to improve treatment strategies for male breast cancer.

The tumor microenvironment in female breast cancer is now recognized as a critical participant in determining the tumor biology. In this environment, the stromal cell-derived factor-1 (SDF1, also known as CXCL12)/the C-X-C chemokine receptor type 4 (CXCR4) axis as well as the hepatocyte growth factor (HGF)/c-MET axis play a role in promoting tumor progression and metastasis, as demonstrated in ex vivo cell experiments and in vivo mouse models of breast cancer [12–14]. The HGF/c-MET axis induces several biological responses in cancer cells, which lead to cell migration, matrix degradation, invasiveness, and induction of angiogenesis [15]. Moreover, overexpression of CXCR4, HGF, and c-MET in primary breast cancer is associated with worse patient outcomes in females [14, 16–20]. Treatments targeting CXCR4 and c-MET in female metastatic breast cancer studied in early-phase clinical trials were tolerated well, and partial response and stable disease were observed [21–24].

However, whether the CXCL12/CXCR4 and the HFG/c-MET axis hold similar significance in male breast cancer is unknown. These microenvironment factors are of interest because the host/environment in male breast cancer will likely be different from female breast cancer.

In order to gain more insight into the male breast cancer tumor and environment biology, we studied a large male breast cancer cohort from The Netherlands. This cohort is part of the international EORTC 10085/TBCRC/BIG/NABCG International Male Breast Cancer Program (NCT01101425). We aimed to explore the tumor expression of HGF, c-MET, CXCL12, and CXCR4 and their correlation with patient overall survival (OS).

## Methods

### Patients

The EORTC 10085/TBCRC/BIG/NABCG International Male Breast Cancer Program (NCT01101425) was launched in 2006. This program is a global effort that aims to improve understanding of the biology of male breast cancer and to optimize its clinical management. An important part of this program was to retrospectively analyze male breast cancer tissue of patients diagnosed between 1990 and 2010 in 93 centers in nine countries. A total of 1800 male patients with invasive breast cancer and an age above 18 years at the time of diagnosis, were eligible and enrolled in the main study. The present substudy analyzed the data of the 841 Dutch patients included. This cohort was identified through the Netherlands Cancer Registry. Patient, treatment, and tumor characteristics were collected from the EORTC database. In the tumor, ER, progesterone receptor (PR), androgen receptor (AR), HER2 and Ki67 expression, histological subtype, grade, and lymphovascular invasion had been previously centrally reviewed [25, 26]. Definitions for positivity of ER, PR, AR, and HER2, and breast cancer subtype surrogate characterization were reported earlier for all 1800 enrolled patients [26]. Briefly, ER, PR, and AR were reported by Allred scores, with positivity defined as a score ≥ 3 and high positivity as a score of 7 or 8. HER2 status was determined according to the American Society of Clinical Oncology-College of American Pathologist (ASCO-CAP) guidelines [26]. Breast cancer subtype surrogates were characterized according to the 2013 St. Gallen consensus guideline, where the low level of Ki67 expression was reported as the percentage of positive cells < 20% and high level of Ki67 expression as ≥ 20% [26]. The archival tissue of all patients was handled according to the Dutch Code for Proper Use of Human Tissue (www.fedara.org). According to the Dutch Central Committee on Research involving Human Subjects, this retrospective non-interventional study did not require informed consent from these patients.

### Tissue microarray construction and immunohistochemistry

Paraffin-embedded primary breast cancer tissue was retrospectively collected by the “Borstkanker Onderzoek Groep” (BOOG). For each formalin-fixed paraffin-embedded (FFPE) block, three representative cores were selected and taken to construct tissue microarrays (TMA) using an Automated Tissue Arrayer ATA-27 (Beecher Instruments, Inc.) [27]. Four-micrometer-thick tissue slides were cut from these TMA blocks for immunohistochemical staining of HGF, c-MET, CXCL12, and CXCR4.

Immunohistochemical staining was performed in one batch per marker to prevent intensity differences. Positive control slides determined with primary antibodies and negative control slides with immunoglobulin class-matched control sera were included on the liver for HGF, female breast cancer tissue for c-MET, rectum for CXCL12, and kidney for CXCR4. Besides this, we also included intestine, heart, brain, liver, lung, stomach, kidney, pancreas, placenta, muscle, testis, and tonsil tissues in each of the TMA slides as the internal control. These tissues express different levels of the studied markers, supporting the specificity of the antibodies. Heat-mediated antigen retrieval was performed with a microwave in a citrate buffer (10 mM citrate, pH 6.0) for CXCL12 and Tris-EDTA buffer (pH 8.0) for HGF and c-MET. Antigen retrieval was not performed for CXCR4 staining. Endogenous peroxidase was blocked with 0.3% H_2_0_2_ in phosphate-buffered saline (PBS; Cl_2_H_3_K_2_Na_3_O_8_P_2_, pH 7.4). A specific binding was blocked with human AB serum. Primary antibodies (anti-HGF [28]: AF-294-NA [10 μg/ml], R&D Systems; anti-c-MET [29]: ab51067 [1.269 μg/ml], anti-CXCR4 [30]: ab10403 [5 μg/ml], anti-CXCL12 [31]: ab25117 [10 μg/ml], all Abcam) were diluted in PBS supplemented with 1% bovine serum albumin. Horseradish peroxidase (HRP)-conjugated goat anti-rabbit and HRP-conjugated rabbit anti-goat antibodies (DAKO) were used as secondary and tertiary antibodies respectively for CXCL12 and c-MET staining. HRP-conjugated rabbit anti-goat and HRP-conjugated goat anti-rabbit antibodies were used as secondary and tertiary antibodies (DAKO), respectively, for HGF staining. Staining was visualized using 3,3′-diaminobenzidine and hematoxylin counterstaining.

The immunohistochemistry slides were digitized with a Digital Slide Scanner NanoZoomer and were viewed with NDP software (Hamamatsu, Japan). Only the patients who had two or more cores containing tumor and stromal cells were included for analysis. Two observers, blinded for the clinicopathological characteristics of patients, scored the digitalized images (SQ and JvR) with the supervision of a dedicated breast pathologist (BvdV). CXCL12, HGF, and c-MET staining was scored using a 0–2 scale (0, no staining; 1+, weak staining; 2+, strong staining), as was the percentage of tumor cells stained per intensity. Subsequently, *H*-scores were calculated for each marker by combining the percentage and intensity (formula used: 1 × percentage of cells with weak staining + 2 × percentage of cells with strong staining). CXCR4 staining was scored as the percentage of tumor cells with a positive nuclear and with a cytoplasm staining, as the intensity of CXCR4 staining was too homogeneous to use the 0–2 scale. The percentages or *H*-scores from two observers were averaged to obtain the score for each core. In case of discrepancy (defined as > 20% difference in percentage or *H*-score), a third observer who was blinded to the scores obtained from the two observers re-scored the cores. For these discrepant cores, a consensus score obtained from SQ and JvR was used, based on the scores from the three observers. The average percentage or *H*-score of replicate cores was used as the final score for each patient. The median percentage or *H*-score of each studied marker was used as the cutoff to define low and high expression. The studied markers were also expressed in some stromal cells, such as the fibroblasts. However, the staining intensity of the markers in stromal cells was far weaker than their staining intensity in the tumor cells. Therefore, in this study, we did not explore the prognostic value of the studied markers expressed in the stromal cells.

### Statistical analysis

The categorical variables were described by percentages, and continuous variables by median and interquartile range (IQR).

OS was defined as the time between the date of diagnosis and the documented date of death due to any cause. The remaining patients were censored at the last date known to be alive. OS was only defined within the subset of M0 patients at diagnosis, as the sample size of M1 patient group was too small to draw conclusions. Patients with unknown metastastic status (Mx) at the time of diagnosis were also excluded from the OS analysis, as the metastastic status largely influence the patient survival. Only patients with non-missing status/dates of survival were used for the OS analysis. The prognostic value of the markers was determined using univariable and multivariable Cox regression analysis. Variables with a *P* value of less than 0.1 in the univariable analysis were included in the multivariable analysis. We used the listwise deletion method for handling missing data. In this method, an entire sample was excluded from the analysis if any single value is missing for the variables used in the multivariable Cox regression analysis. For the studied markers which were not associated with patient survival in the univariable analysis, their prognostic values were further investigated in the preplanned subgroup analyses. OS was analyzed using the Kaplan-Meier method, with a log-rank test assessing its difference. All tests and *P* values tested two-sided were considered significant when ≤ 0.05. Statistical analysis was performed using the Statistical Package for the Social Sciences (SPSS) version 19.0 (SPSS. Inc.).

## Results

### Patient and tumor characteristics

**Fig. 1:**
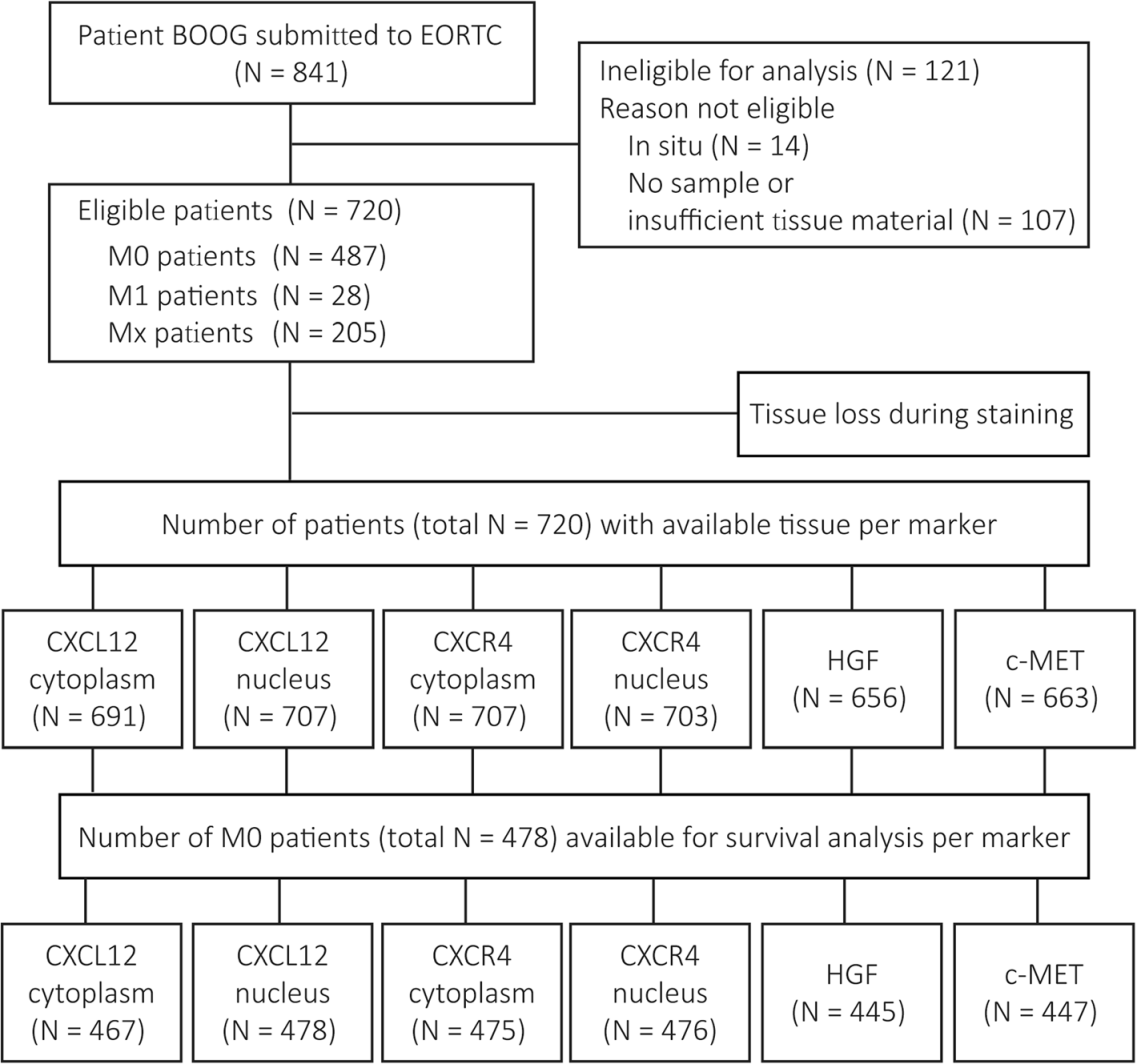
CONSORT diagram of the studied patients, number of patients with available tissue for each marker, and number of M0 patients per marker available for survival analysis. BOOG, Borstkanker Onderzoek Groep; EORTC, European Organisation for Research and Treatment of Cancer; CXCR4, C-X-C chemokine receptor type 4; CXCL12, C-X-C motif chemokine 12; HGF, hepatocyte growth factor; M0, patients without metastases at diagnosis; M1, patients with metastases at diagnosis; Mx, patients with unknown metastatic status at diagnosis

In 720 out of the 841 patients, sufficient tumor tissue was available for analysis (Fig. 1). Patient and tumor characteristics at the time of diagnosis of these 720 patients are shown in Table 1. The median age at diagnosis of breast cancer was 67 years (IQR, 58–76 years). Almost all patients had ER-positive tumors (98.3%), with 91.0% highly positive. PR positivity was observed in 76.8% and AR positivity in 96.9% of cases. HER2 positivity was present in tumors of 31 patients (4.9%). The majority of patients had a luminal-like breast cancer subtype, with 43.3% luminal A-like and 49.9% luminal B-like HER2−. There were 487 (67.6%) patients that were free from metastasis (M0) at the time of diagnosis. The treatment information of these 487 patients is provided in Additional file 1. The median follow-up for the M0 patients was 6.5 years (range, 0.04–23.8).

**Table 1:** Patient and tumor characteristics of 720 male breast cancer patients with sufficient tumor tissue available for analysis

Characteristics	No. (%)	% exclude missing
**Age at diagnosis**		
Median (IQR)	67 (58–76) years	
**ER (Allred score)**		
0–2	11 (1.5)	(1.7)
3–6	47 (6.6)	(7.3)
7–8	584 (81.1)	(91.0)
Missing	78 (10.8)	
**PR (Allred score)**		
0–2	146 (20.3)	(23.2)
3–6	252 (35.0)	(40.0)
7–8	232 (32.2)	(36.8)
Missing	90 (12.5)	
**AR (Allred score)**		
0–2	20 (2.8)	(3.1)
3–6	62 (8.7)	(9.8)
7–8	556 (77.2)	(87.1)
Missing	82 (11.4)	
**HER2 status**		
Negative	597 (82.9)	(94.0)
Positive	31 (4.3)	(4.9)
Equivocal	7 (1.0)	(1.1)
Missing	85 (11.8)	
**Ki67**		
≤ 20%	502 (69.7)	(79.1)
> 20%	133 (18.5)	(20.9)
Missing	85 (11.8)	
**Breast cancer subtypes (2013 St. Gallen consensus)**		
Luminal A-like	270 (37.5)	(43.3)
Luminal B-like HER2−	311 (43.2)	(49.9)
Luminal B-like HER+	31 (4.3)	(5.0)
HER2+ (non-luminal)	0 (0)	(0)
Triple-negative	9 (1.3)	(1.4)
Not defined (ER−, PR+)	2 (0.3)	(0.3)
Missing	97 (13.5)	
**Histological type**		
Invasive ductal	628 (87.2)	(88.3)
Invasive lobular	9 (1.3)	(1.3)
Others	74 (10.3)	(10.4)
Missing	9 (1.3)	
**Histological grade**		
I	165 (22.9)	(23.2)
II	373 (51.8)	(52.5)
III	172 (23.9)	(24.2)
Missing	10 (1.4)	
**Metastatic status at diagnosis**		
M0	487 (67.6)	(94.6)
M1	28 (3.9)	(5.4)
Mx	205 (28.5)	

*AR* androgen receptor, *ER* estrogen receptor, *HER2* human epidermal growth factor receptor 2, *IQR* interquartile range, *M0* no metastasis, *M1* with metastasis, *Mx* metastatic status unknown, *PR* progesterone receptor

### Expression of the studied markers in the primary tumor

**Fig. 2:**
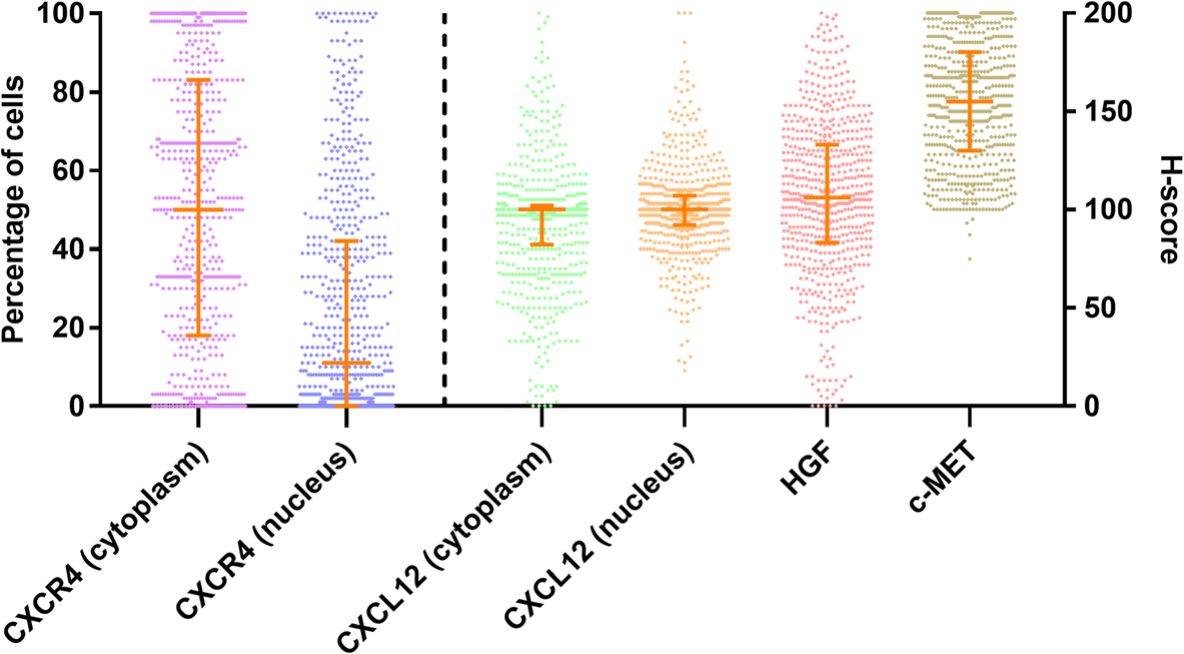
The expression of the studied markers in male breast cancer as assessed by immunohistochemistry. CXCR4 is presented as the percentage of cells with positive staining; CXCL12, HGF, and c-MET are presented as *H*-score. Each dot represents data for an individual patient. The orange line indicates the median and interquartile range. The dotted line separates markers on its left side presented as the percentage and markers on its right side presented as the *H*-score. CXCR4, C-X-C chemokine receptor type 4; CXCL12, C-X-C motif chemokine 12; HGF, hepatocyte growth factor

The exact number of tumors tested per marker is shown in Fig. 1. Representative images of positive or negative expression for CXCR4, and negative, weak, or strong staining for CXCL12, HGF, and c-MET are shown in Additional file 2. The concordance rates of the percentage/*H*-score for each TMA core (defined as ≤ 20% difference in percentage or *H*-score) among the studied markers between the two observers are shown in Additional file 3. The percentage of positive cells for CXCR4 expression and *H*-score for CXCL12, HGF, and c-MET are demonstrated in Fig. 2. The median CXCR4 expression per tumor was 50% (IQR, 18–83%) in the cytoplasm and 11% (IQR, 0–42%) in the nucleus. The median *H*-score for the CXCL12 expression in the cytoplasm was 100 (IQR, 83–102) and 100 (IQR, 92–107) for the nucleus expression. The median *H*-scores for HGF and c-MET expression were 106 (IQR, 83–133) and 155 (IQR, 130–180), respectively. Information on the heterogeneity of the studied markers between the cores is provided in Additional files 4, 5, 6, and 7.

### Prognostic value of the studied markers for OS in M0 patients

**Fig. 3:**
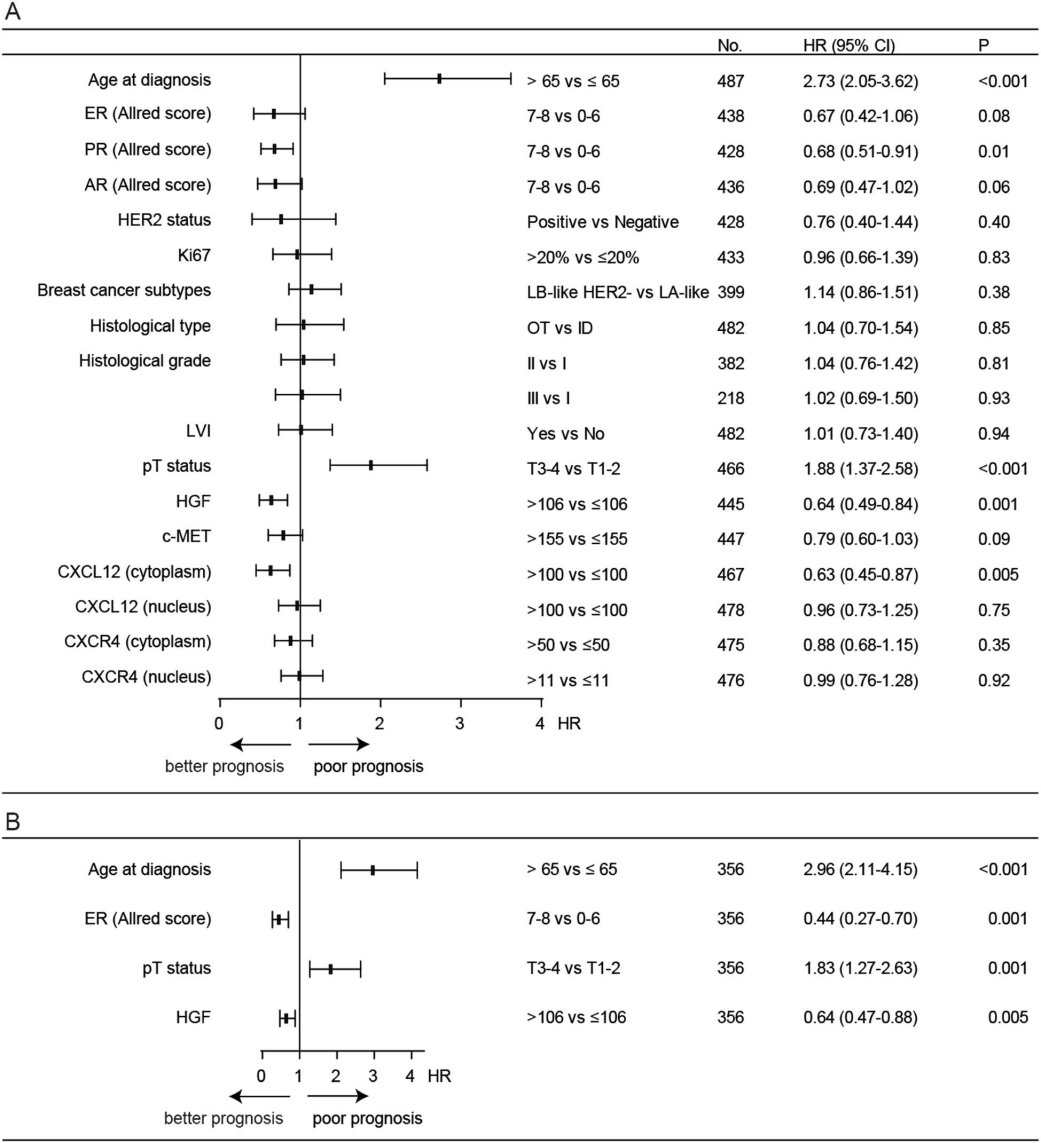
Factors associated with overall survival in M0 patients at diagnosis in univariable (**a**) and multivariable (**b**) Cox regression analysis. **a** HGF, CXCL12 (cytoplasm), age at diagnosis, PR, and pT status are statistically significantly associated with overall survival. **b** HGF, age at diagnosis, ER, and pT status are independent predictors for overall survival. AR, androgen receptor; CI, confidence interval; CXCR4, C-X-C chemokine receptor type 4; CXCL12, C-X-C motif chemokine 12; ER, estrogen receptor; HER2, human epidermal growth factor receptor 2; HGF, hepatocyte growth factor; HR, hazard ratio; ID, invasive ductal; LVI, lymphovascular invasion; M0, no metastasis; PR, progesterone receptor; pT status, pathological tumor status; OT, other types, including invasive lobular, mixed, micropapillary, mucinous, cribriform, tubular, metaplastic, clear cell, and apocrine carcinoma

**Fig. 4:**
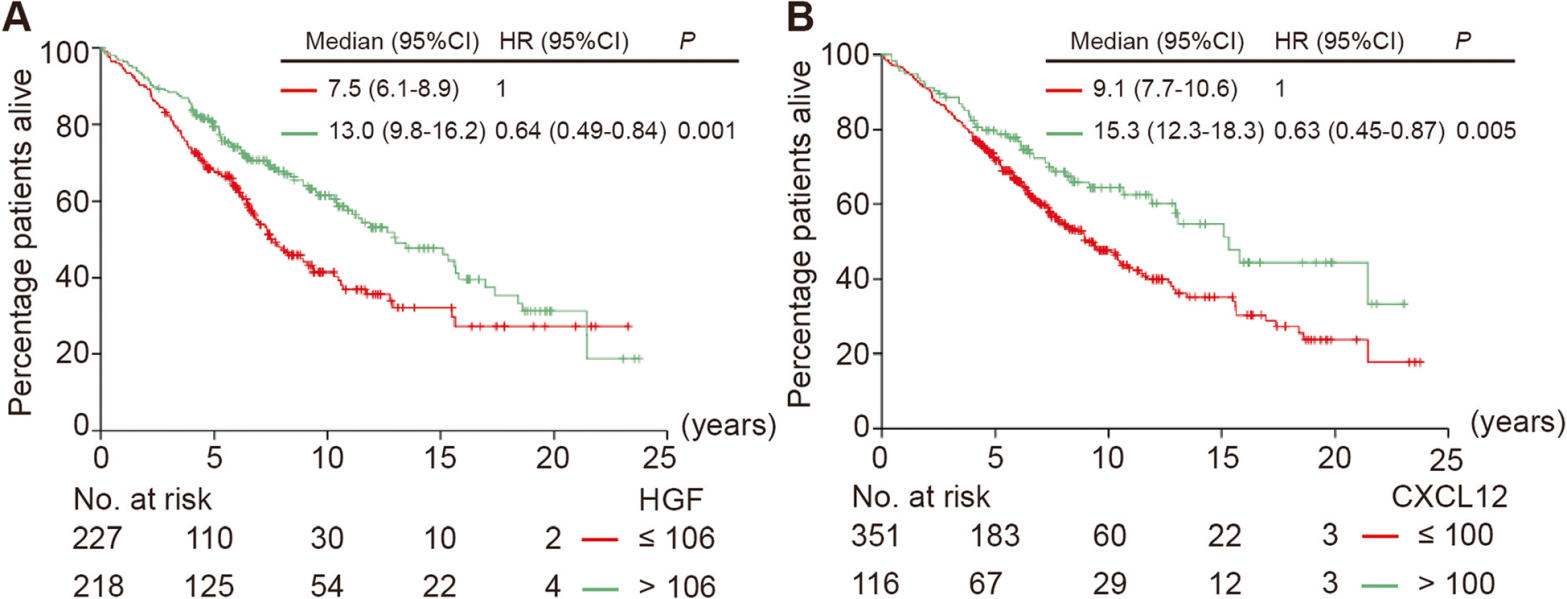
Kaplan-Meier analysis for the overall survival of M0 patients at diagnosis classified by HGF and CXCL12 (cytoplasm) tumor expression. CXCL12, C-X-C motif chemokine 12; HGF, hepatocyte growth factor; M0, no metastasis

**Fig. 5:**
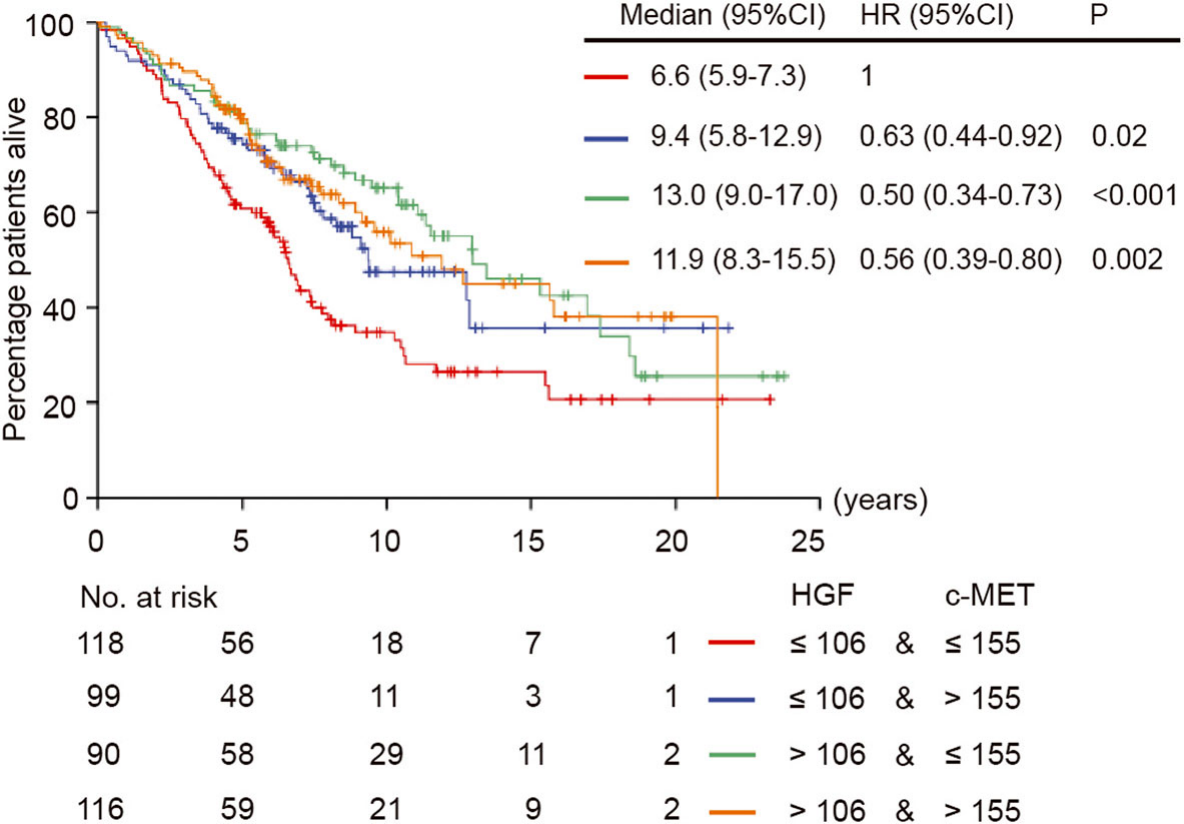
Kaplan-Meier analysis for overall survival of M0 patients at diagnosis classified by HGF and c-MET tumor expression. CI, confidence interval; HGF, hepatocyte growth factor; HR, hazard ratio; M0, no metastasis

The exact number of M0 patients with available data for survival analysis is shown in Fig. 1. Both HGF and CXCL12 (cytoplasm) expression by tumor cells were correlated to OS in univariable Cox regression analysis, and HGF remained significant in the multivariable analysis (Fig. 3). Median OS was 7.5 years (95% CI, 6.1–8.9) for patients with HGF low-expressing tumors and 13.0 years (95% CI, 9.8–16.2) for those with HGF high-expressing tumors [hazard ratio (HR), 0.64 (95% CI, 0.49–0.84), *P* = 0.001]. Median OS was 9.1 years (95% CI, 7.7–10.6) for patients with CXCL12 (cytoplasm) low-expressing tumors and 15.3 years (95% CI, 12.3–18.3) for those with CXCL12 (cytoplasm) high-expressing tumors [HR, 0.63 (95% CI, 0.45–0.87), *P* = 0.005] (Fig. 4). In the subgroup analysis, c-MET was correlated to OS in the subgroups with patients older than 65 years at diagnosis, PR low-expressing tumors, luminal B-like HER2− breast cancer subtype, invasive ductal tumors, and histological grade II tumors (see Additional file 8). CXCL12 (nucleus), CXCR4 (cytoplasm), and CXCR4 (nucleus) were not associated with OS in any patient or tumor subgroup in the univariable analysis (see Additional files 9, 10, and 11). Based on these findings, we further classified patients according to the expression of HGF and c-MET. The median OS of patients with both HGF and c-MET low-expressing tumors was 6.6 years (95% CI, 5.9–7.3), which was shorter than the OS of the other subgroups (Fig. 5). Age at diagnosis, PR, and pT status were the other parameters associated with OS (Fig. 3).

## Discussion

In this unique cohort of male breast cancer patients, we identified HGF expression in the primary tumor to be an independent predictor for better OS in the non-metastatic setting. In addition, high expression of CXCL12 in the cytoplasm of tumor cells in the primary tumor was associated with better OS.

Remarkably, the prognostic value of HGF and c-MET is contradictory to the findings in female breast cancer. In female breast cancer, HGF and c-MET have been identified as a predictor for worse outcomes and are associated with a high Ki-67 labeling index [14, 16–19]. This led to the hypothesis that HGF acts as a mitogen in female breast cancer [32]. In our cohort in male breast cancer, we identified HGF to be an independent prognostic factor for better OS.

The prognostic value of the CXCL12 expression in male breast cancer is comparable with the findings in female breast cancer reported in the literature [33, 34]. In female breast cancer, the cytoplasmic expression of CXCL12 was associated with better disease-free survival and OS [33, 34]. These results are confirmed by a recently published meta-analysis, which included 8 studies with a total of 2205 patients [35]. Four of these studies (*N* = 953) measured the CXCL12 protein expression which was positively correlated with disease-free survival and OS.

The remarkable difference in the prognostic value of HGF and c-MET compared to female breast cancer might result from differences in tumor and environment biology between male and female breast cancer. This is in line with a recent study of DNA sequencing analysis on 1943 cancer-related genes in 135 patients with male breast cancer, which demonstrated differences in the genomic landscape between male and female breast cancers. Somatic mutations in homologous recombination deficiency-related genes were more prevalent in male breast cancer compared to female breast cancer, whereas TP53 somatic mutations were less frequent [36]. Currently, it becomes clear that some important markers in breast cancer biology can play a different role in male compared to female breast cancer. When dependency patterns of key oncoproteins were compared between 134 male and 728 female breast cancer tissues, some similar patterns were observed for both genders, such as p53 and hypoxia-inducible factor 1-alpha [8]. However, also clear differences were identified. For example, the expression of PR showed in female breast cancer a continuous dependency on cytokeratin 8/18, cyclin D1, B cell lymphoma 2 (Bcl-2), and cyclin-dependent kinase inhibitor p21 [8]. In male breast cancer, however, PR showed no dependency on these markers, indicating that PR is subject to effects from other markers [8]. AR had a stronger effector function in males compared to female tumors [8]. Results from the 21-gene breast recurrence score, used to characterize the molecular features of breast cancer, also indicate distinct differences in male compared to female breast cancer. Men below 40 years of age had a higher recurrence score compared to females while above 60 years men had a larger proportion with a low recurrence score [10]. Therefore, although differences between male and female breast cancer become apparent, the crosstalk among predominant biologic pathways and their function in males is not well understood, including that of the HGF/c-MET signaling.

In female breast cancer, HGF/c-MET promotes cell proliferation, migration, and invasion by HGF binding-induced c-MET activation of the phosphoinositide 3-kinase/Akt pathway and the Erk/mitogen-activated protein kinase cascade [14]. Furthermore, high expression of HGF or c-MET was associated with higher histological tumor grade and worse patient outcomes [16, 19]. In the present study, higher expression of both HGF and c-MET was associated with improved OS. One possible factor contributing to this difference might be the age difference between male and female breast cancer patients. Preclinical evidence suggests that with advancing age, the tumor stroma exhibits alterations, such as decreased interferon signaling and antigen presentation. These changes may influence the proliferative effects of the tumor microenvironment [37]. The influence of age on the tumor microenvironment needs to be further elucidated but might lead to new insight into the dynamics of the tumor microenvironment.

Our study has limitations. First, due to its retrospective nature, data of some patient and tumor characteristics are missing. Nevertheless, the number of M0 patients excluded for the OS analysis was limited, and therefore, a significant impact on our findings appears unlikely. Second, currently, there is no widely accepted standardized methodology for immunohistochemical staining and the scoring of the studied markers. This may create bias in interpreting the data. These issues can be addressed in the prospective Male Breast Cancer Program prospective part (NCT01101425), which has finalized the inclusion and of which analyses are ongoing.

## Conclusions

In the present study, HGF and CXCL12 tumor expression identified male breast cancer patients with good prognosis. Whether this insight provides possible options for intervention strategies should be determined in future studies.

## Supplementary information

**Additional file 1: Table S1.** Pattern of treatment for 487 patients without metastasis at diagnosis.

**Additional file 2: Figure S1.** Examples of negative and positive staining of CXCR4; negative, weak (1+) and strong (2+) staining of CXCL12, HGF and c-MET by immunohistochemistry. Abbreviations: CXCL12, C-X-C motif chemokine 12; CXCR4, C-X-C chemokine receptor type 4; HGF, hepatocyte growth factor.

**Additional file 3: Table S2.** The concordance rates of the percentage/H-score for each TMA core among the studied markers between the two observers.

**Additional file 4.** Information on the heterogeneity of percentage of positive cells for CXCR4 between the TMA cores.

**Additional file 5.** Information on the heterogeneity of H-score for CXCL12 between the TMA cores.

**Additional file 6.** Information on the heterogeneity of H-score for HGF between the TMA cores.

**Additional file 7.** Information on the heterogeneity of H-score for c-MET between the TMA cores.

**Additional file 8: Figure S2.** Subgroup analysis by patient and tumor characteristics of the prognostic value of c-MET for overall survival in patients without metastasis. c-MET is associated with overall survival in the subgroups with patients older than 65 years at diagnosis, PR low expression tumors, Luminal B-like HER2- breast cancer subtype, invasive ductal tumors and histological grade II tumors. Abbreviations: AR, androgen receptor, CI, confidence interval; ER: estrogen receptor; HR, hazard ratio; PR, progestrone receptor; pT status: pathological tumor status.

**Additional file 9: Figure S3.** Subgroup analysis by patient and tumor characteristics of the prognostic value of CXCL12 (nucleus) for overall survival in patients without metastasis. CXCL12 (nucleus) is not associated with overall survival in any subgroup. Abbreviations: AR, androgen receptor, CI, confidence interval; CXCL12, C-X-C motif chemokine 12; ER: estrogen receptor; HR, hazard ratio; PR, progestrone receptor; pT status: pathological tumor status.

**Additional file 10: Figure S4.** Subgroup analysis by patient and tumor characteristics of the prognostic value of CXCR4 (cytoplasm) for overall survival in patients without metastasis. CXCR4 (cytoplasm) is not associated with overall survival in any subgroup. Abbreviations: AR, androgen receptor, CI, confidence interval; CXCR4, C-X-C chemokine receptor type 4; ER: estrogen receptor; HR, hazard ratio; PR, progestrone receptor; pT status: pathological tumor status.

**Additional file 11: Figure S5.** Subgroup analysis by patient and tumor characteristics of the prognostic value of CXCR4 (nucleus) for overall survival in patients without metastasis. CXCR4 (nucleus) is not associated with overall survival in any subgroup. Abbreviations: AR, androgen receptor, CI, confidence interval; CXCR4, C-X-C chemokine receptor type 4; ER: estrogen receptor; HR, hazard ratio; PR, progestrone receptor; pT status: pathological tumor status.

## Data Availability

The datasets generated during and/or analyzed during the current study are available from the corresponding author on reasonable request.

## Abbreviations

AR: Androgen receptor
ASC0-CAP: American Society of Clinical Oncology-College of American Pathologist
Bcl-2: B cell lymphoma 2
BOOG: Borstkanker Onderzoek Groep
CXCL12 (SDF1): Stromal cell-derived factor-1
CXCR4: C-X-C chemokine receptor type 4
ER: Estrogen receptor
FFPE: Formalin-fixed paraffin-embedded
HER2: Human epidermal growth factor receptor 2
HGF: Hepatocyte growth factor
HR: Hazard ratio
HRP: Horseradish peroxidase
IQR: Interquartile range
OS: Overall survival
PBS: Phosphate-buffered saline
PR: Progesterone receptor
SPSS: Statistical Package for the Social Sciences
TMA: Tissue microarrays
