# Supplementary material for: High hepatocyte growth factor expression in primary tumor predicts better overall survival in male breast cancer
Source: Breast Cancer Res. 2020 Mar 18;22:30. doi: 10.1186/s13058-020-01266-x (PMC7081628; doi:10.1186/s13058-020-01266-x)
Supplement: Supplementary file 1 — Additional file 1: Table S1. Pattern of treatment for 487 patients without metastasis at diagnosis. [file 13058_2020_1266_MOESM1_ESM.docx]

Additional file 1

| Table S1. Pattern of treatment for 487 patients without metastasis at diagnosis | | |
| --- | --- | --- |
| Characteristics | No (%) | % exclude missing |
| **Breast surgery** |  |  |
| No surgery | 0 (0) | (0) |
| Breast-conserving surgery | 11 (2.3) | (4.3) |
| (Modified) radical mastectomy | 243 (49.9) | (95.7) |
| Missing | 233 (47.8) |  |
| **Management of regional nodes** |  |  |
| No surgery | 10 (2.1) | (4.0) |
| SLNB | 64 (13.1) | (25.5) |
| ALND +/- SLNB | 177 (36.3) | (70.5) |
| Missing | 236 (48.5) |  |
| **Adjuvant radiotherapy** |  |  |
| No | 145 (29.8) | (57.8) |
| Yes | 106 (21.8) | (42.8) |
| Missing | 236 (48.4) |  |
| **(Neo)adjuvant chemotherapy** |  |  |
| No | 192 (39.4) | (76.2) |
| Yes | 60 (12.3) | (23.8) |
| Missing | 235 (48.3) |  |
| If yes, (neo)adjuvant chemotherapy regimen |  |  |
| CMF | 7 (11.7) | (12.1) |
| Anthracycline based | 37 (61.7) | (63.8) |
| Anthracycline and taxanes | 12 (20.0) | (20.7) |
| Other | 2 (3.3) | (3.4) |
| Missing | 2 (3.3) |  |
| **Adjuvant trastuzumab** |  |  |
| No | 248 (50.9) | (98.0) |
| Yes | 5 (1.0) | (2.0) |
| Missing | 234 (48.0) |  |
| **Adjuvant endocrine therapy** |  |  |
| No | 107 (22.0) | (43.3) |
| Yes | 140 (28.7) | (56.7) |
| Missing | 240 (49.3) |  |
| If yes, specify planned treatment |  |  |
| Tamoxifen | 112 (80.0) |  |
| Aromatase inhibitor (AI) | 5 (3.6) |  |
| Tamoxifen followed by AI | 10 (7.1) |  |
| Tamoxifen + LHRH | 11 (7.9) |  |
| AI + LHRH | 0 (0) |  |
| Other | 2 (1.4) |  |
| SLNB: sentinel lymph node biopsy; ALND: axillary lymph node dissection; LHRH: luteinizing hormone releasing hormone | | |
