# Supplementary figures and images for: High hepatocyte growth factor expression in primary tumor predicts better overall survival in male breast cancer
Source: Breast Cancer Res. 2020 Mar 18;22:30. doi: 10.1186/s13058-020-01266-x (PMC7081628; doi:10.1186/s13058-020-01266-x)

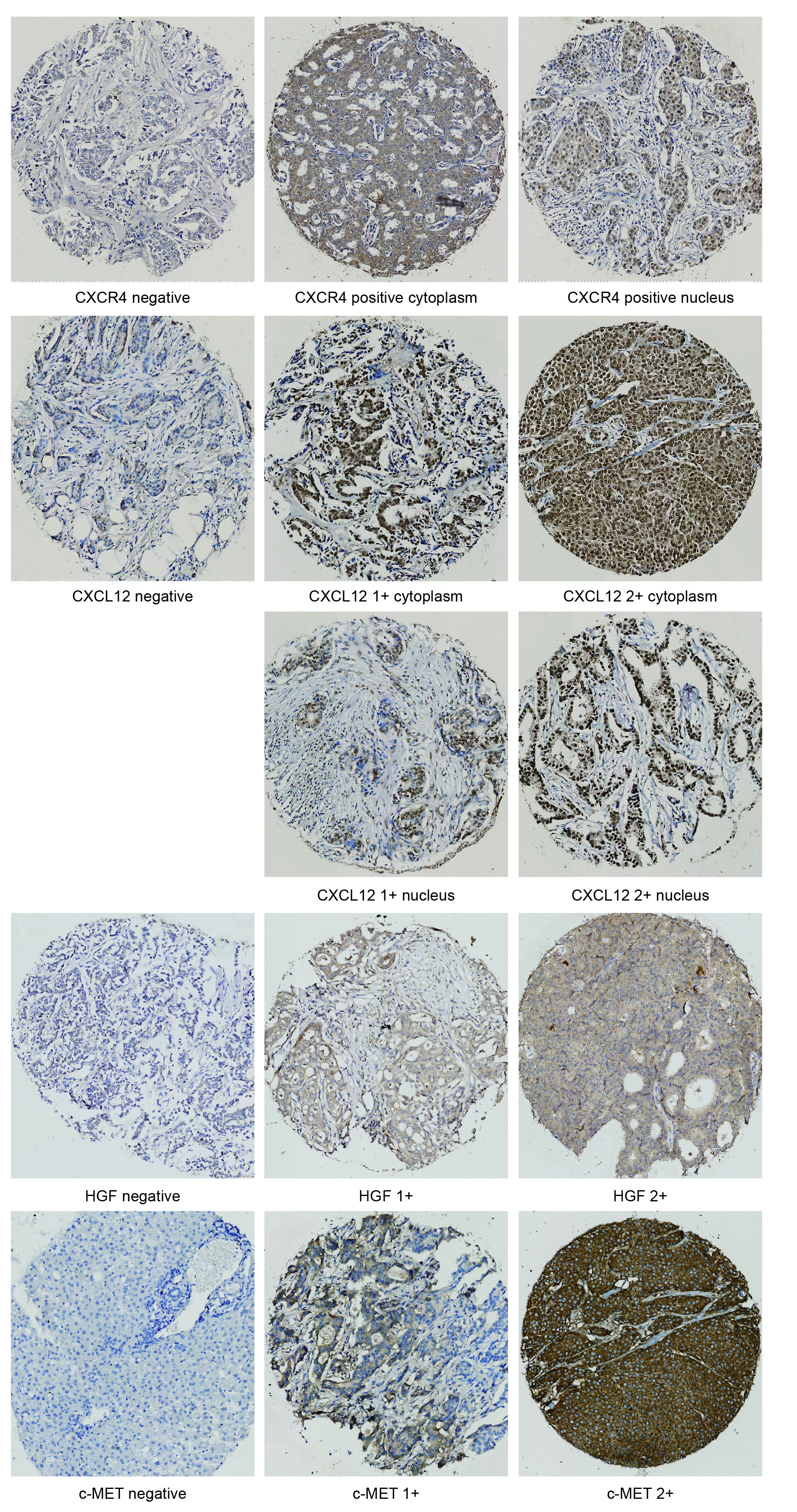

Supplement: Supplementary file 2 — Additional file 2: Figure S1. Examples of negative and positive staining of CXCR4; negative, weak (1+) and strong (2+) staining of CXCL12, HGF and c-MET by immunohistochemistry. Abbreviations: CXCL12, C-X-C motif chemokine 12; CXCR4, C-X-C chemokine receptor type 4; HGF, hepatocyte growth factor. [file 13058_2020_1266_MOESM2_ESM.jpg]

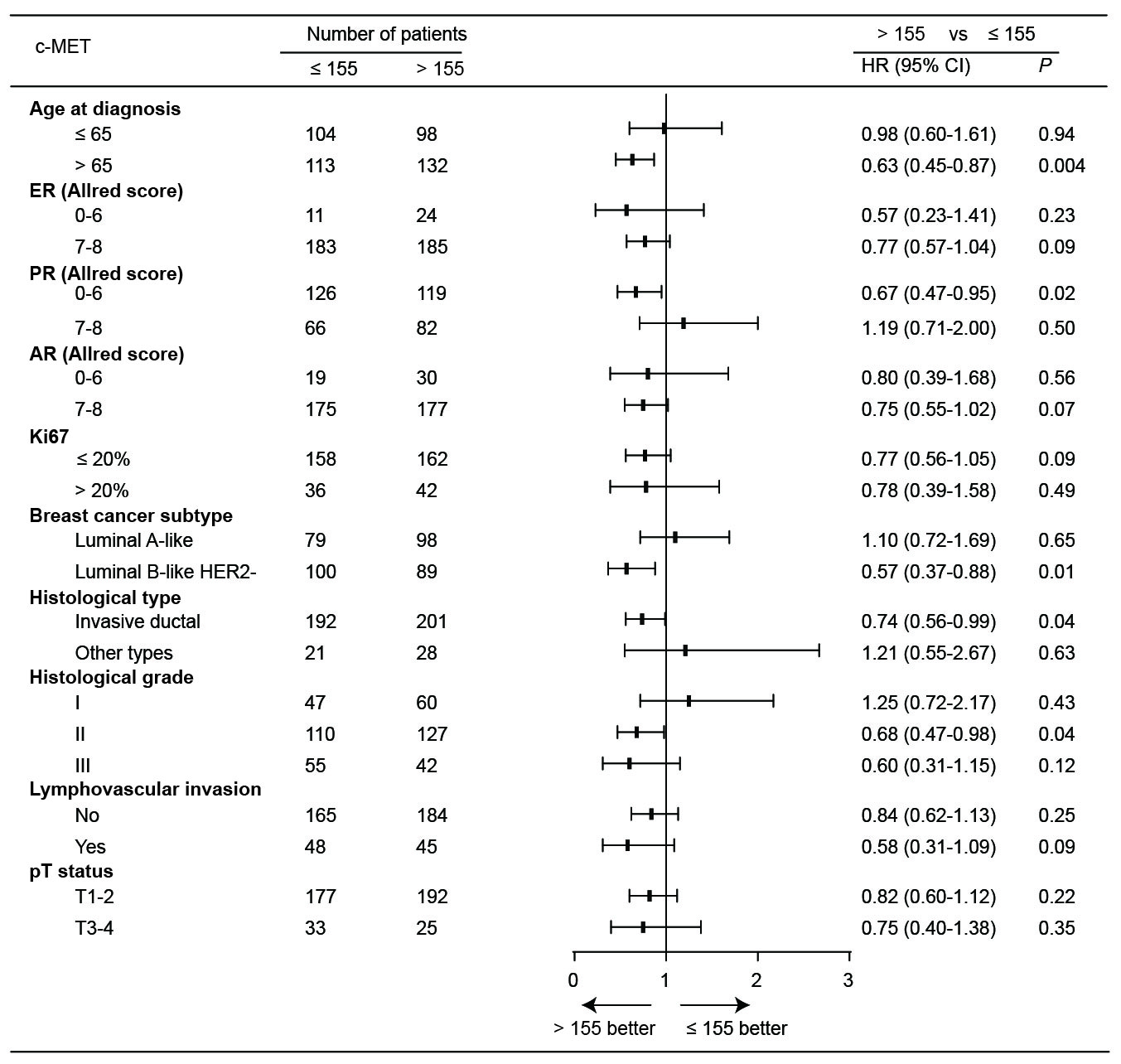

Supplement: Supplementary file 8 — Additional file 8: Figure S2. Subgroup analysis by patient and tumor characteristics of the prognostic value of c-MET for overall survival in patients without metastasis. c-MET is associated with overall survival in the subgroups with patients older than 65 years at diagnosis, PR low expression tumors, Luminal B-like HER2- breast cancer subtype, invasive ductal tumors and histological grade II tumors. Abbreviations: AR, androgen receptor, CI, confidence interval; ER: estrogen receptor; HR, hazard ratio; PR, progestrone receptor; pT status: pathological tumor status. [file 13058_2020_1266_MOESM8_ESM.jpg]

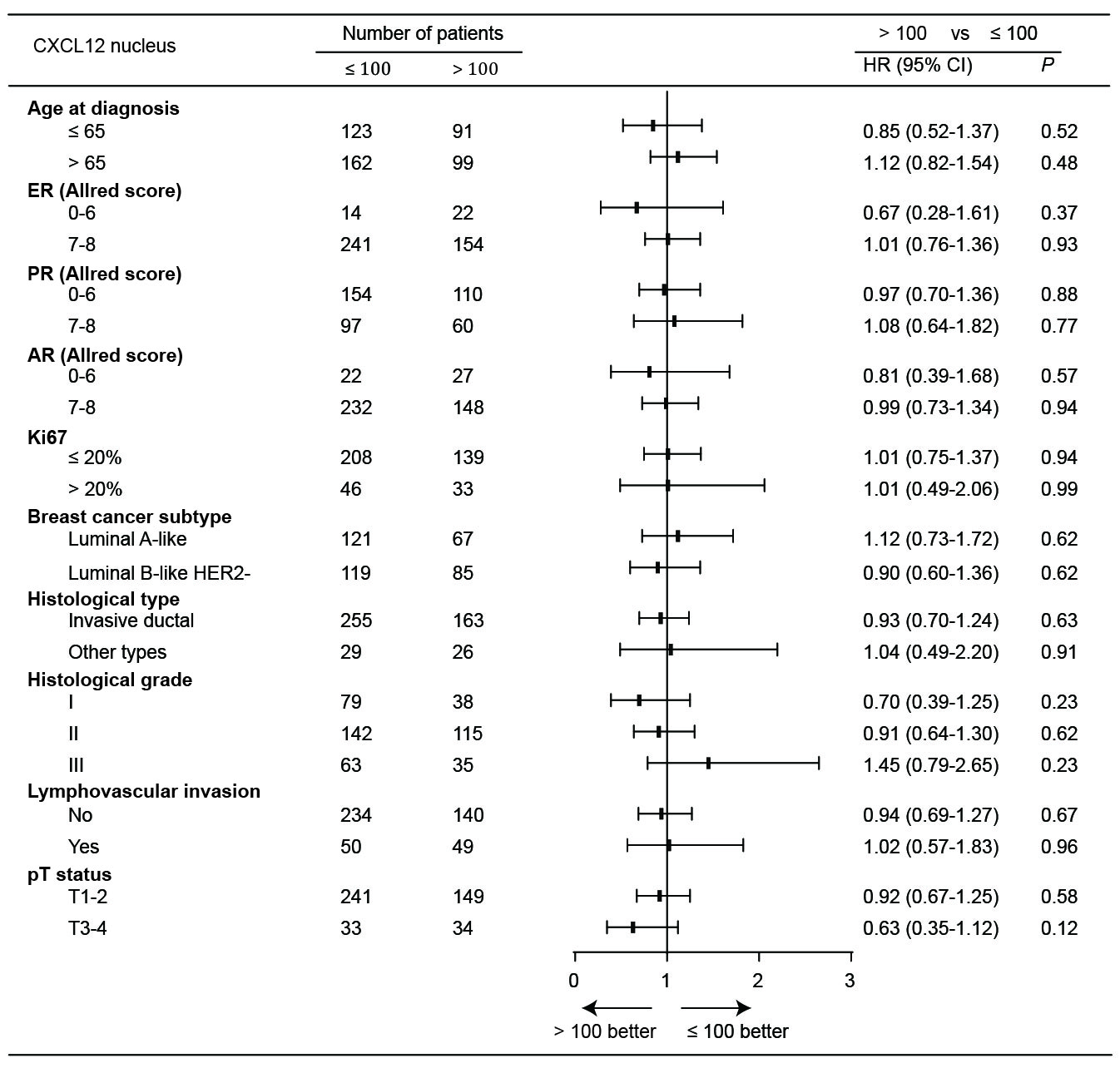

Supplement: Supplementary file 9 — Additional file 9: Figure S3. Subgroup analysis by patient and tumor characteristics of the prognostic value of CXCL12 (nucleus) for overall survival in patients without metastasis. CXCL12 (nucleus) is not associated with overall survival in any subgroup. Abbreviations: AR, androgen receptor, CI, confidence interval; CXCL12, C-X-C motif chemokine 12; ER: estrogen receptor; HR, hazard ratio; PR, progestrone receptor; pT status: pathological tumor status. [file 13058_2020_1266_MOESM9_ESM.jpg]

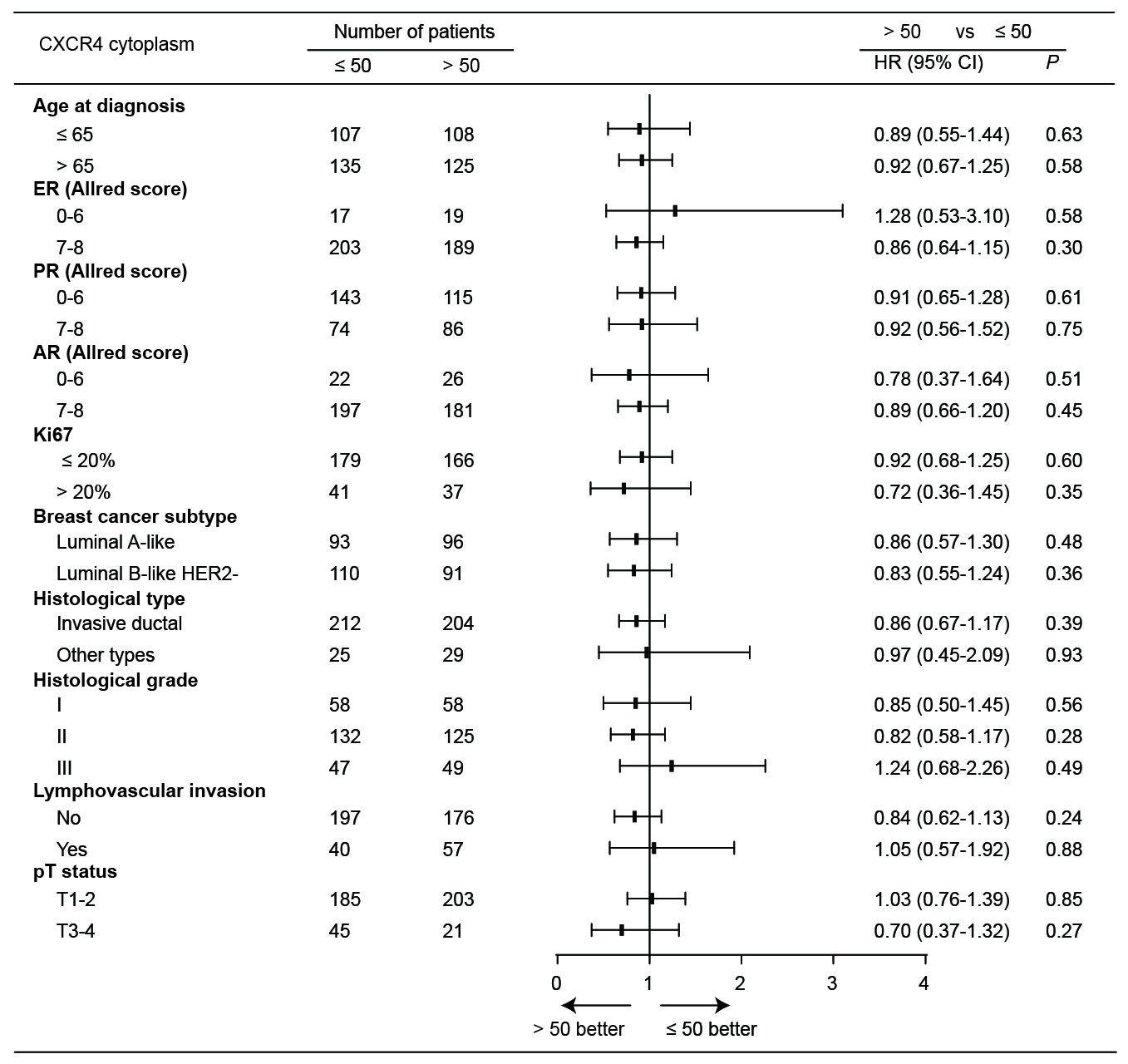

Supplement: Supplementary file 10 — Additional file 10: Figure S4. Subgroup analysis by patient and tumor characteristics of the prognostic value of CXCR4 (cytoplasm) for overall survival in patients without metastasis. CXCR4 (cytoplasm) is not associated with overall survival in any subgroup. Abbreviations: AR, androgen receptor, CI, confidence interval; CXCR4, C-X-C chemokine receptor type 4; ER: estrogen receptor; HR, hazard ratio; PR, progestrone receptor; pT status: pathological tumor status. [file 13058_2020_1266_MOESM10_ESM.jpg]

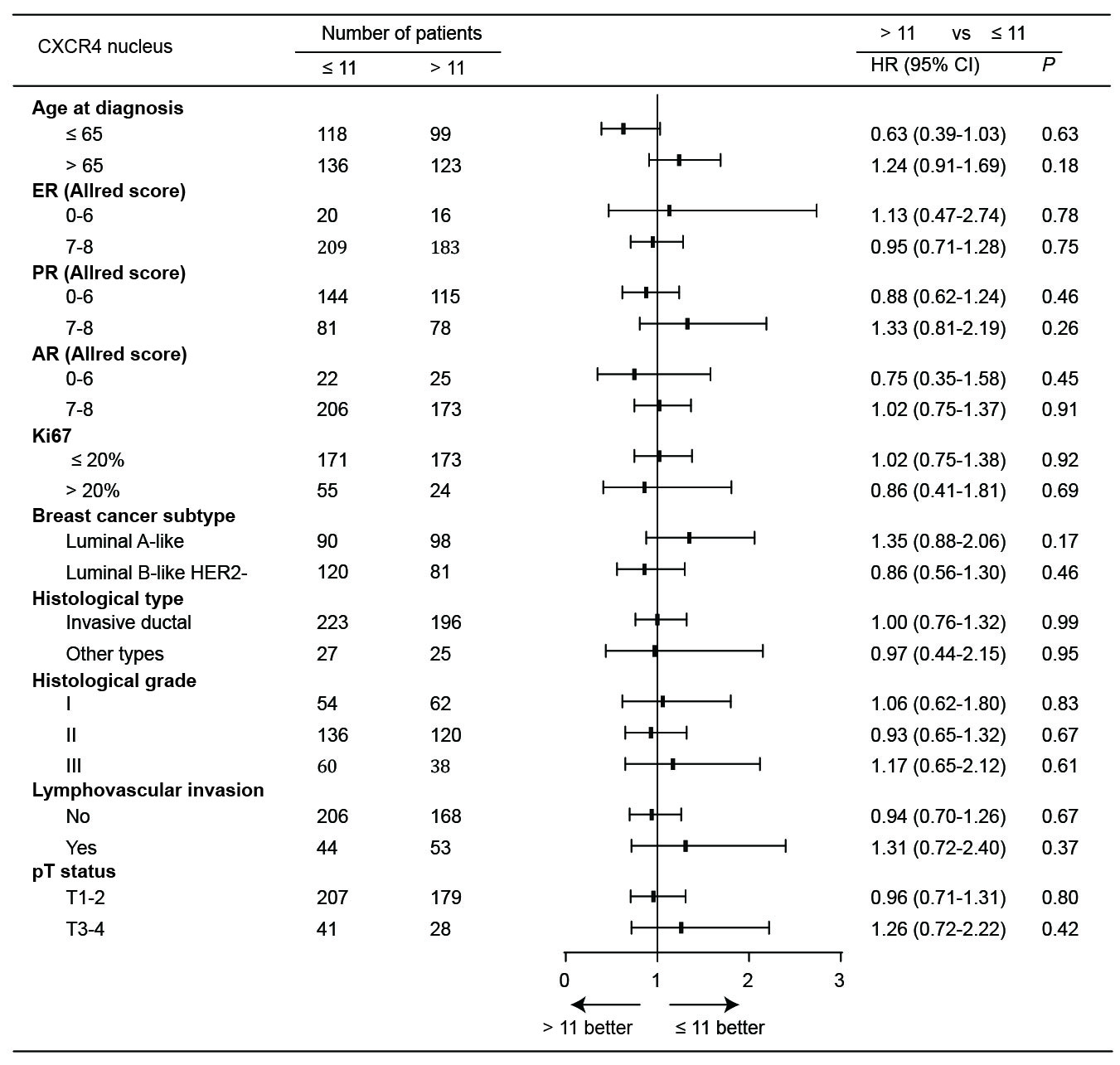

Supplement: Supplementary file 11 — Additional file 11: Figure S5. Subgroup analysis by patient and tumor characteristics of the prognostic value of CXCR4 (nucleus) for overall survival in patients without metastasis. CXCR4 (nucleus) is not associated with overall survival in any subgroup. Abbreviations: AR, androgen receptor, CI, confidence interval; CXCR4, C-X-C chemokine receptor type 4; ER: estrogen receptor; HR, hazard ratio; PR, progestrone receptor; pT status: pathological tumor status. [file 13058_2020_1266_MOESM11_ESM.jpg]
