# Supplementary material for: High hepatocyte growth factor expression in primary tumor predicts better overall survival in male breast cancer
Source: Breast Cancer Res. 2020 Mar 18;22:30. doi: 10.1186/s13058-020-01266-x (PMC7081628; doi:10.1186/s13058-020-01266-x)
Supplement: Supplementary file 3 — Additional file 3: Table S2. The concordance rates of the percentage/H-score for each TMA core among the studied markers between the two observers. [file 13058_2020_1266_MOESM3_ESM.docx]

Additional file 3

| Table S2. The concordance rates* of the percentage/H-score for each TMA core among the studied markers between the two observers. | |
| --- | --- |
| Studied markers | Concordance rate (%) |
| CXCR4 cytoplasm | 68 |
| CXCR4 nucleus | 82 |
| CXCL12 cytoplasm | 77 |
| CXCL12 nucleus | 86 |
| HGF | 51 |
| c-MET | 60 |
| CXCL12 (also known as SDF1): stromal cell-derived factor-1; CXCR4: C-X-C chemokine receptor type 4; HGF: hepatocyte growth factor; TMA: tissue microarray; *: defined as ≤ 20% difference in percentage or H-score. | |
